# Ring-opening metathesis of some strained bicyclic systems; stereocontrolled access to diolefinated saturated heterocycles with multiple stereogenic centers

**DOI:** 10.3762/bjoc.14.247

**Published:** 2018-10-24

**Authors:** Zsanett Benke, Melinda Nonn, Márton Kardos, Santos Fustero, Loránd Kiss

**Affiliations:** 1 Institute of Pharmaceutical Chemistry, University of Szeged, Eötvös u. 6, H-6720 Szeged, Hungary; 2 MTA-SZTE Stereochemistry Research Group, Hungarian Academy of Sciences, Eötvös u. 6, H-6720 Szeged, Hungary; 3 Departamento de Química Orgánica, Facultad de Farmàcia, Universidad de Valencia, Av. Vicente Andrés Estellés, s/n 46100 Valencia, Spain

**Keywords:** functionalization, heterocycles, metathesis, ring opening, stereogenic centers

## Abstract

Ring-opening metathesis (ROM) of various unsaturated, constrained bicyclic ring systems has been investigated with the use of commercial ruthenium-based catalysts. Starting from various cyclodienes, the corresponding derived bicyclic lactone, lactam, and isoxazoline derivatives were submitted to ROM under ethenolysis. These functionalized, strained bicyclic systems afforded novel highly-functionalized diolefinated heterocyclic scaffolds in ROM reactions with stereocontrol, through the conservation of the configuration of the stereogenic centers of the starting compounds.

## Introduction

Metathesis reactions, among them ring-opening metathesis (ROM), have received a great deal of attention in synthetic organic chemistry, affording access to various highly functionalized, alkenylated molecular entities [1–10].

Highly functionalized three-dimensional organic scaffolds with multiple stereogenic centers as small molecular entities represent an important segment of organic and pharmaceutical chemistry. Therefore, selective syntheses with stereocontrol of such scaffolds [11–12], such as highly-functionalized olefinated derivatives [13], are of main importance and a major challenge in synthetic organic chemistry. Thus, ring-opening metathesis is a powerful and widely applied methodology for the synthesis of such derivatives, including alkenylated molecular scaffolds with multiple stereogenic centers [14–16] and references cited therein. Diversity-oriented synthesis (DOS), with the aim of the preparation of structurally diverse elements of small molecules, has become increasingly important in drug research, and well recognized as a common approach to generate molecular libraries. Results with respect to the various strategies utilized in DOS with special focus on selective and stereocontrolled methods have been published [17–20]. The major features of these studies are the use of readily available and easily accessible starting materials towards the construction of diverse and complex scaffolds and the application of the resulting compound collections in drug discovery.

Since their ring C–C double bond offers a number of possible chemical transformations, cyclic dienes with different ring sizes might be considered to be important starting materials for the generation of structurally diverse molecules. Among the large number of possible transformations, the ring olefinic bond of alicyclic dienes may lead to valuable β-lactams [21–23] or γ-lactams [24], shown to be highly important precursors for the access of various structures (e.g., amino acids, azido esters, hydroxylated amino esters, fluorinated amino esters, etc.) with various functional groups as well as stereochemical and skeletal diversity [21–23].

## Results and Discussion

**Scheme 1:**
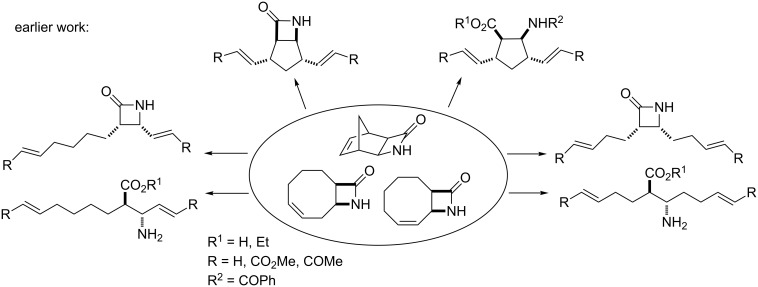
ROM of various bicyclic unsaturated β-lactams [14–16].

Recently, we have demonstrated the high utility of various constrained cyclic dienes, such as norbornadiene as well as 1,5- and 1,3-cyclooctadienes in the context of their applicability towards the access of diverse, highly functionalized olefinated molecules [14–16]. The corresponding β-lactams derived from cyclodienes were used as starting substances for further functionalization with ROM. We have described a stereocontrolled synthetic route to access difunctionalized cyclic β-amino acid derivatives [14] and β-lactams [15–16] based on ring-opening metathesis (ROM) through ethenolysis of the structurally restricted cycloalkene β-amino acids or unsaturated bicyclic β-lactams, followed by cross-coupling metathesis (CM) of the newly created C–C double bonds (Scheme 1).

**Scheme 2:**
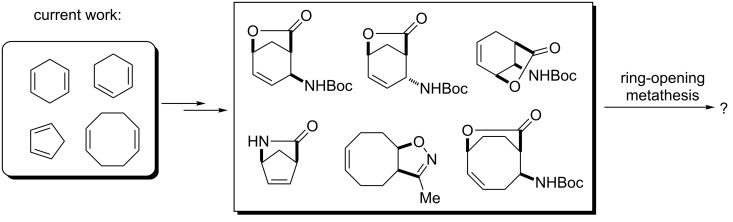
ROM of various constrained bicyclic unsaturated systems (γ-lactones, δ-lactones, γ-lactam, isoxazoline).

Our current goal was to expand the study of the ROM protocol of functionalized strained ring systems to the investigation of functionalized derivatives such as bicyclic lactones, γ-lactams or isoxazolines, derived from various cyclodienes and to evaluate their chemical behavior under Ru-catalyzed ring-opening conditions (Scheme 2).

**Figure 1:**
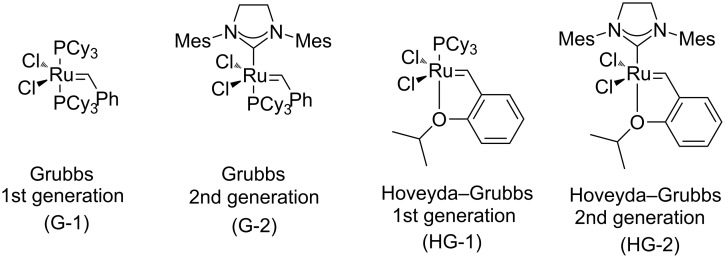
Commercial Ru-based catalysts used in the current work.

**Scheme 3:**
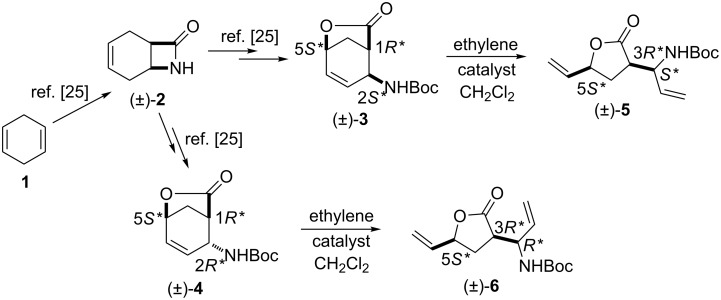
ROM of lactones (±)-**3** and (±)-**4**.

First, the ring opening of racemic bicyclic γ-lactone (±)-**3** (derived from cyclodiene **1** via β-lactam (±)-**2**) [25] was investigated. Ring opening was performed in ethylene atmosphere at 20 °C in the presence of four commercially available Ru-based catalysts (5 mol %, Figure 1). Note that based on our earlier results [15], bicyclic unsaturated lactam (±)-**2** bearing the azetidinone ring fused with a six-membered ring system thus possessing ring strain, did not afford any ROM products. Interestingly, lactone (±)-**3** in the presence of second generation catalysts (G-2 and HG-2) provided the corresponding ring-opened compound (±)-**5** albeit with modest yields (Scheme 3, Table 1).

**Table 1:** Isolated yields for compound (±)-**5** formed in the ring-opening reaction of lactone (±)-**3** with ethylene in ROM reactions with various catalysts.

catalyst	G-1 catalyst	G-2 catalyst	HG-1 catalyst	HG-2 catalyst

product				

(±)-**5**	0%	21%	0%	25%

In the presence of G-2 and HG-2 catalysts, bicyclic lactone (±)-**4** a stereoisomer of (±)-**3** furnished olefinated γ-lactone (±)-**6** similar to (±)-**5** (Scheme 3, Table 2). Unfortunately, ROM reactions, however, took place with total conversions, they were always accompanied by the formation of a significant amount of polymeric materials (ROMP) responsible for the observed modest yields of these reactions. Noteworthy, neither the variation of the catalyst loading (amount or in portion) nor the substrate concentration (in 5, 10, 20 or 30 mL of solvent) had any significant influence on the yield of the products.

**Table 2:** Isolated yields for compound (±)-**6** formed in the ring-opening reaction of lactone (±)-**4** with ethylene in ROM reactions with various catalysts.

catalyst	G-1 catalyst	G-2 catalyst	HG-1 catalyst	HG-2 catalyst

product				

(±)-**6**	0%	26%	traces	36%

Next, racemic lactone (±)-**9** (synthesized from 1,3-cyclohexadiene (**7**) through lactam (±)-**8**) [26] was subjected to ring-opening reactions with all four catalysts.

**Scheme 4:**
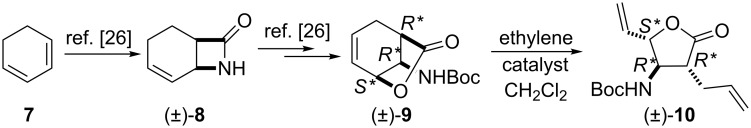
ROM of lactones (±)-**9**.

It should be noted again, that based on our earlier findings [15], bicyclic lactam (±)-**8** did not provide any ring-opened product, while bicyclic lactone (±)-**9** could be opened with G-2 and HG-2 catalysts (5 mol %) affording olefinated amino lactone (±)-**10** at 20 °C. Notably, the yield of the transformation with catalyst HG-2 to obtain lactone derivative (±)-**10** was twice as high as in the case of G-2 (Scheme 4, Table 3).

**Table 3:** Isolated yields for compound (±)-**10** formed in the ring-opening reaction of lactone (±)-**9** with ethylene in ROM reactions with various catalysts.

catalyst	G-1 catalyst	G-2 catalyst	HG-1 catalyst	HG-2 catalyst

product				

(±)-**10**	0%	16%	traces	35%

**Scheme 5:**
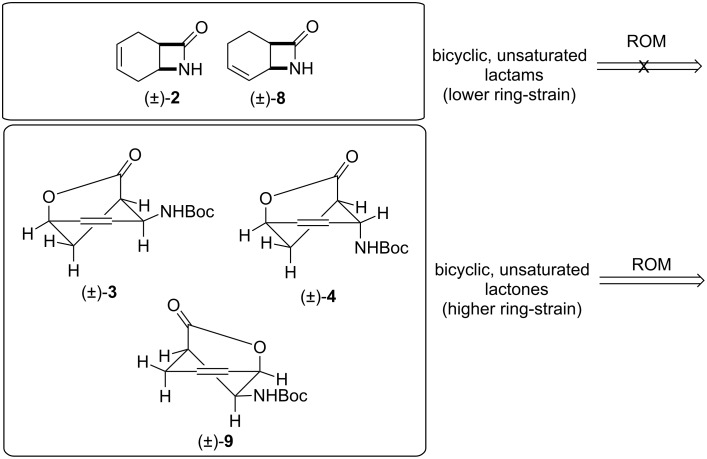
ROM of structurally constrained bicyclic lactones and lactams.

From the above comparative results it may be assumed that unsaturated bicyclic β-lactams (±)-**2** and (±)-**8**, bearing the fused four-membered and six-membered ring system, have a lower ring strain than bicyclic, unsaturated γ-lactones (±)-**3**, (±)-**4** and (±)-**9**. Because of their higher constraint, the latter compounds underwent ring opening providing the corresponding monocyclic, dialkenylated amino lactones, albeit with modest yields (Scheme 5); (for relevant literature date for the ROM for various cyclic systems with ring strain see ref. [27–29].

We continued our ring-opening investigations with other model derivatives possessing a larger ring system. According to results published previously [15] and in contrast with bicyclic cyclohexene-fused lactams (±)-**2** and (±)-**8**, lactam (±)-**12** [30], derived from 1,5-cyclooctadiene, afforded the corresponding dialkenylated ring-opened product under ROM protocol.

**Scheme 6:**
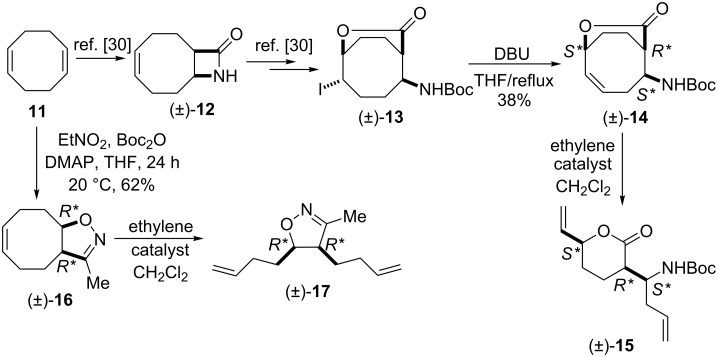
ROM of bridged lactone (±)-**14** and cyclooctene-fused isoxazoline (±)-**16**.

The isolated yields of (±)-**15** were higher than those of the analogous cyclohexene systems in the presence of both G-2 and HG-2 catalysts because of the higher ring strain of the eight-membered framework. Bicyclic, unsaturated bridged lactone (±)-**14** (derived from (±)-**12**) underwent ring-opening not only with second generation catalysts but also with HG-1 (5 mol %), leading at 20 °C to δ-lactone derivative (±)-**15** although with low yield (Scheme 6, Table 4). In continuation, we selected a cyclooctene-fused system, namely isoxazoline (±)-**16** which, in turn, was accessed through nitrile–oxide dipolar cycloaddition, by using nitroethane, DMAP and Boc_2_O.

Ring opening proved to be successful with second generation catalysts, yielding the corresponding diolefinated isoxazoline (±)-**17** (Scheme 6).

**Table 4:** Isolated yields for compounds formed in the ring-opening reaction of lactone (±)-**14** and isoxazoline (±)-**16** with ethylene in ROM reactions with various catalysts.

catalyst	G-1 catalyst	G-2 catalyst	HG-1 catalyst	HG-2 catalyst

product				

(±)-**15**	0%	52%	11%	59%
(±)-**17**	38%	–	–	0%

**Scheme 7:**
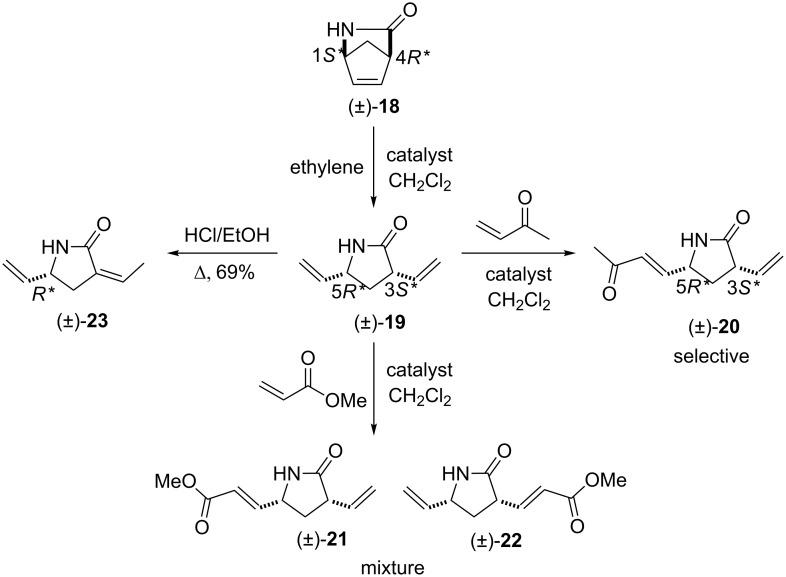
ROM and transformations of lactam (±)-**18**.

Our studies were continued with the ROM reactions of conformationally restricted γ-lactam (±)-**18** (Vince’s lactam) as model compound [24]. The ring opening in ethylene atmosphere of bridged pyrrolidinone (±)-**18** took place at 20 °C and afforded the corresponding divinylated lactam (±)-**19** [31–32]. Somewhat surprisingly, in contrast to model derivatives used previously, the highest yield (70%) was attained with first generation catalyst HG-1 (5 mol %). In the presence of the second generation catalysts, in turn, the ring-opened pyrrolidinone derivative (±)-**19** could be isolated only in low yields (Scheme 7, Table 5).

**Table 5:** Isolated yields for compound (±)-**19** formed in the ring-opening reaction of lactam (±)-**18** with ethylene in ROM reactions with various catalysts.

catalyst	G-1 catalyst	G-2 catalyst	HG-1 catalyst	HG-2 catalyst

product				

(±)-**19**	9%	29%	70%	15%

As observed, the ROM reactions of the investigated unsaturated cyclic substrates (namely (±)-**3**, (±)-**4**, (±)-**9**, (±)-**14**, (±)-**16** and (±)-**18**) gave different results in view of the used Ru-based catalyst, which allowed us to conclude that all these transformations are highly substrate and catalyst dependent, the nature of the structure of the cyclic starting material determining the outcome of the transformations. It is well known that the prediction of the behavior of the catalyst efficiency is a rather difficult task. Metathesis reactions are known to be often catalyst or substrate dependent. Electronic or steric factors, and chelation effects may contribute to the outcome of metathesis in view of the yield. Moreover, possible H-bonding interactions in the intermediate phase between the catalyst chlorine and the substrate may be responsible for the accomplishments of the reactions, which were deeply investigated and discussed in the literature [33–37] and see references therein. In our case it was observed that the imidazole carbene-based catalysts (G-2 and HG-2) were effective in case of bridged lactones with a six-membered ring part in their framework, with O-functionalities (±)-**3**, (±)-**4**, (±)-**9** and (±)-**14**. In case of isoxazoline-fused derivative (±)-**16** G-1 gave the best result, while in case of lactam (±)-**18** HG-1 was the most efficient. The observed results regarding the current ROM processes were somewhat surprising, the overall comparison of these experimental investigations in the ROM may depend strongly on the structure of the substrates.

The valuable dialkenylated compounds (lactones, lactams, isoxazolines) with multiple stereogenic centers thus synthesized can be considered interesting scaffolds for further transformations in view of the access of novel three-dimensional functionalized scaffolds through cross-metathesis (CM). An illustrative example is shown on Scheme 7. Divinylated γ-lactam (±)-**19** selected as a model compound was first subjected to CM with methyl acrylate. When the reaction was performed in the presence of Ru-based catalysts, in CH_2_Cl_2_, either at reflux temperature or at 20 °C, it gave a mixture of monometathesised products ((±)-**21** and (±)-**22**) after 6 h together with a large amount of polymeric materials.

**Figure 2:**
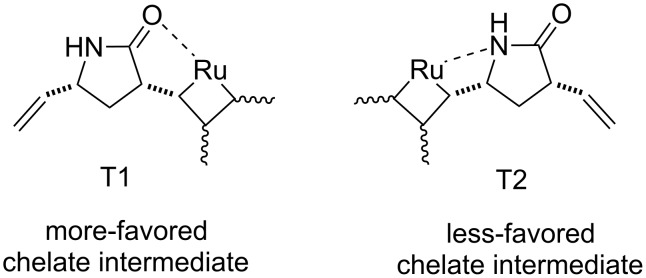
Chelate intermediates in CM of (±)-**19**.

The products could not be separated by means of chromatography. Interestingly, however, the CM of (±)-**19** with methyl vinyl ketone induced by G-2, HG-1 or HG-2, afforded a single derivative, monometathesised compound (±)-**20** bearing the oxo group closest to the amide N-atom (Scheme 7, Table 6). Compound (±)-**20** was formed in low yields and *E*-selectively with the chemodiscrimination of the olefinic bonds. The observed low yields for the formation of (±)-**20** might be explained by stereoelectronic factors. The coordinating ability of both the O- and N-atom of the amide with the Ru atom in the metallacyclobutane intermediate may reduce the reactivity of the olefinic bonds. Furthermore, the chelating ability of the amide heteroatoms is also assumed to be responsible for the chemodiscrimination of the vinyl groups. Namely, the chelating five-membered structure **T1** is more favored than **T2** and, therefore, the vinyl group closest to the ring N-atom becomes more reactive in cross-metathesis (Figure 2).

**Table 6:** Isolated yields for compound (±)-**20** formed in the reaction of lactam (±)-**19** in CM reactions with various catalysts.

				
catalyst	G-1 catalyst	G-2 catalyst	HG-1 catalyst	HG-2 catalyst

product				

(±)-**20**	0%	5%	19%	28%

Similar chemodiscriminations of C–C double bonds were previously observed in the transformation of various alkenylated lactams or amino esters [16]. Lactams are known to be useful precursors for the preparation of amino acids and amino esters [21–22]. When compound (±)-**19** was subjected to either acid-catalyzed hydrolysis or ethanolysis at reflux, it furnished a pyrrolidinone derivative identified as (±)-**23**, instead of the expected product (amino acids or amino ester) formed via the opening of the heteroring, (Scheme 7). The process involves isomerization through olefin bond migration proceeding *Z*-selectively.

## Conclusion

The ring-opening metathesis (ROM) of some ring-constrained, unsaturated bicyclic frameworks has been studied in the presence of commercially available ruthenium-based catalysts. The bicyclic systems, derived from various cyclodienes, such as lactone, lactam or isoxazoline derivatives, were investigated under ROM through ethenolysis, which afforded novel dialkenylated scaffolds formed under stereocontrol with the conservation of the configuration of the stereogenic centers. The resulting diolefinated aminolactones, isoxazolines or lactam derivatives with multiple stereogenic centers might be considered to be interesting highly-functionalized three-dimensional compounds for further derivatizations. Extensions of the ROM of various bicyclic, conformationally restricted derivatives are currently being studied by our group.

## Experimental

### General procedure for the ring-opening metathesis

To a solution of bicyclic olefin derivative (150 mg) in anhydrous CH_2_Cl_2_ (20 mL) the catalyst (5 mol %) was added (see Tables) and the mixture was stirred at 20 °C in the presence of an ethylene atmosphere for the time indicated in the text (monitored by TLC). After completion of the reaction, the mixture was concentrated under vacuum and purified by column chromatography on silica gel (*n*-hexane/EtOAc).

### General procedure for cross-metathesis

To a solution of γ-lactam derivative (80 mg) in anhydrous CH_2_Cl_2_ (15 mL), catalyst (5 mol %, see Table) and methyl vinyl ketone or methyl acrylate (4 equiv) were added and the mixture was stirred for the time and temperature indicated in text. After completion of the reaction (monitored by TLC), the mixture was concentrated under vacuum and the residue was purified by column chromatography on silica gel (*n*-hexane/EtOAc).

### General procedure for the nitrile–oxide cycloaddition

To a solution of 1,5-cyclooctadiene (1.5 mmol) in THF (20 mL), EtNO_2_ (5 equiv), DMAP (0.3 mmol, 20 mol %) and Boc_2_O (4.5 mmol, 3 equiv) were added and the mixture was stirred at 20 °C for 24 h. The reaction mixture was then diluted with H_2_O (30 mL) and extracted with EtOAc (3 × 15 mL). The combined organic layer was washed with brine (2 × 20 mL), dried (Na_2_SO_4_) and concentrated under vacuum. The crude residue was purified by column chromatography on silica gel (*n*-hexane/EtOAc).

#### Characterization of the synthesized substances

*tert*-Butyl ((S*)-1-((3R*,5S*)-2-oxo-5-vinyltetrahydrofuran-3-yl)allyl)carbamate ((±)-**5).**


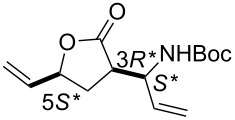


Yellow oil; yield 25%; *R*_f_ 0.70 (*n*-hexane/EtOAc 2:1); ^1^H NMR (CDCl_3_, 400 MHz) δ 1.41 (s, 9H, *t*-Bu), 1.82–1.88 (m, 1H, CH_2_), 2.40–2.47 (m, 1H, CH_2_), 2.98–3.06 (m, 1H, H-3), 4.33–4.39 (m, 1H, CHN), 4.78–4.84 (m, 1H, H-5), 5.23–5.32 (m, 4H, CH=), 5.66–5.82 (m, 3H, CH= and NH); ^13^C NMR (CDCl_3_, 100 MHz) δ 29.0, 29.7, 44.7, 52.5, 79.4, 80.1, 118.7, 119.3, 134.8, 135.1, 155.1, 174.2; MS (ESI, pos) (*m*/*z*): 288 [M + 1], 168 [M − Boc]; anal. calcd for C_14_H_21_NO_4_: C, 62.90; H, 7.92; N, 5.24; found, C, 62.55; H, 7.58; N, 4.89.

*tert*-Butyl ((R*)-1-((3R*,5S*)-2-oxo-5-vinyltetrahydrofuran-3-yl)allyl)carbamate ((±)-**6).**


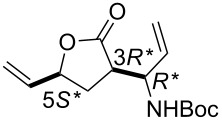


Yellow oil; yield 36%; *R*_f_ 0.72 (*n*-hexane/EtOAc 2:1); ^1^H NMR (CDCl_3_, 400 MHz) δ 1.47 (s, 9H, *t*-Bu), 1.94–1.99 (m, 1H, CH_2_), 2.46–2.51 (m, 1H, CH_2_), 3.00–3.09 (m, 1H, H-3), 4.48–4.54 (m, 1H, CNH), 4.73–4.85 (m, 2H, H-5 and NH), 5.27–5.33 (m, 3H, CH=), 5.40–5.46 (m, 1H, CH=), 5.77–6.01 (m, 2H, CH=); ^13^C NMR (CDCl_3_, 100 MHz) δ 28.9, 29.4, 45.7, 52.0, 79.0, 80.1, 116.8, 118.6, 135.2, 135.7, 155.6, 175.7; MS (ESI, pos) (*m*/*z*): 288 [M + 1], 168 [M − Boc]; anal. calcd for C_14_H_21_NO_4_: C, 62.90; H, 7.92; N, 5.24; found, C, 62.59; H, 8.30; N, 4.87.

*tert-*Butyl ((2S*,3R*,4R*)-4-allyl-5-oxo-2-vinyltetrahydrofuran-3-yl)carbamate ((±)-**10).**


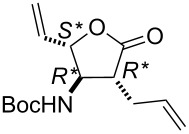


Yellow oil; yield 35%; *R*_f_ 0.70 (*n*-hexane/EtOAc 2:1); ^1^H NMR (CDCl_3_, 400 MHz) δ 1.48 (s, 9H, *t*-Bu), 2.42–2.49 (m, 1H, CH_2_), 2.53–2.58 (m, 1H, CH_2_), 2.61–2.67 (m, 1H, H-4), 3.91–3.97 (m, 1H, H-3), 4.52–4.62 (m, 2H, H-2 and NH), 5.06–5.12 (m, 2H, CH=), 5.33–5.38 (m, 1H, CH=), 5.42–5.48 (m, 1H, CH=), 5.75–5.85 (m, 2H, CH=); ^13^C NMR (CDCl_3_, 100 MHz) δ 18.9, 22.7, 29.4, 45.7, 57.3, 82.4, 118.8, 119.4, 133.1, 133.2, 154.7, 174.3; MS (ESI, pos) (*m*/*z*): 288 [M + 19], 168 [M – Boc]; anal. calcd for C_14_H_21_NO_4_: C, 62.90; H, 7.92; N, 5.24; found, C, 62.59; H, 7.60; N, 4.86.

*tert-*Butyl ((1R*,2S*,6S*,*Z*)-8-oxo-7-oxabicyclo[4.2.2]dec-4-en-2-yl)carbamate ((±)-**14).**


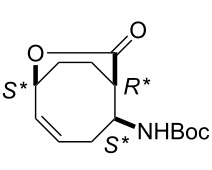


White solid; yield 38%; mp 101–102 °C; *R*_f_ = 0.50 (*n*-hexane/EtOAc 2:1); ^1^H NMR (CDCl_3_, 400 MHz) δ 1.40 (s, 9H, *t-*Bu), 1.68–1.75 (m, 1H, CH_2_), 1.83–1.99 (m, 2H, CH_2_), 2.28–2.35 (m, 2H, CH_2_), 1.42–1.50 (m, 1H, CH_2_), 3.02–3.06 (m, 1H, H-1), 3.90–3.99 (m, 1H, H-2), 5.00–5.08 (brs, 1H, NH), 5.10–5.15 (m, 1H, H-6), 5.47–5.53 (m, 1H, H-4), 5.83–5.92 (m, 1H, H-5); ^13^C NMR (CDCl_3_, 100 MHz) δ 21.4, 25.3, 28.4, 46.8, 55.6, 78.7, 79.8, 125.9, 129.0, 154.6, 173.0; MS (ESI, pos) (*m*/*z*): 288 [M + 1], 168 [M – Boc]; anal. calcd for C_14_H_21_NO_4_: C, 62.90; H, 7.92; N, 5.24; found, C, 63.22; H, 7.59; N, 4.88.

*tert*-Butyl ((S*)-1-((3R*,6S*)-2-oxo-6-vinyltetrahydro-2*H*-pyran-3-yl)but-3-en-1-yl)carbamate ((±)-**15).**


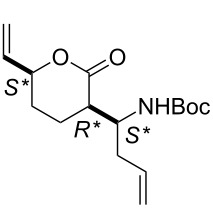


White solid; yield 59%; mp 64–65 °C; *R*_f_ 0.65 (*n*-hexane/EtOAc 2:1); ^1^H NMR (CDCl_3_, 400 MHz) δ 1.42 (s, 9H, *t-*Bu), 1.67–1.74 (m, 2H, CH_2_), 1.93–2.02 (m, 2H, CH_2_), 2.32–2.42 (m, 2H, CH_2_), 2.74–2.81 (m, 1H, H-3), 3.78–3.85 (m, 1H, CHN), 4.81–4.86 (m, 1H, CH=), 5.13–5.10 (m, 2H, CH=), 5.25–5.35 (m, 2H, CH=), 5.38 (brs, 1H, NH), 5.69–5.80 (m, 2H, CH=); ^13^C NMR (CDCl_3_, 100 MHz) δ 20.5, 26.3, 27.0, 35.0, 44.0, 51.1, 78.4, 79.2, 117.4, 117.5, 135.5, 135.7, 155.6, 172.8; MS (ESI, pos) (*m*/*z*): 296 [M + 1]; anal. calcd for C_16_H_25_NO_4_: C, 65.06; H, 8.53; N, 4.74; found, C, 64.69; H, 8.19; N, 4.39.

(3aR*,9aR*,*Z*)-3-Methyl-3a,4,5,8,9,9a-hexahydrocycloocta[*d*]isoxazole ((±)-**16).**


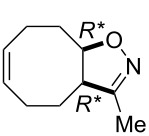


Yellow oil; yield: 62%; *R*_f_ 0.37 (*n*-hexane/EtOAc 4:1); ^1^H NMR (CDCl_3_, 400 MHz): δ 1.80–1.87 (m, 2H, H-4), 1.95 (s, 3H, CH_3_), 2.02–2.19 (m, 3H, H-5, H-8), 2.21–2.36 (m, 1H, H-8), 2.4–2.54 (m, 1H, H-9), 2.97–3.06 (q, 1H, *J*^1^ = 8.64 Hz, *J*^2^ = 8.46 Hz, *J*^3^ = 8.64 Hz, H-3a), 4.37–4.45 (m, 1H, H-9a), 5.55–5.64 (m, 1H, H-6), 5.65–5.73 (m, 1H, H-7); ^13^C NMR (DMSO, 125 MHz) δ 12.3, 24.4, 24.7, 25.1, 28.5, 51.0, 83.9, 129.0, 130.7, 160.9; anal. calcd for C_10_H_15_NO: C, 72.69; H, 9.15; N, 8.48; found, C, 72.38; H, 8.80; N, 8.11.

(4R*,5R*)-4,5-Di(but-3-enyl)-3-methyl-4,5-dihydroisoxazole ((±)-**17).**


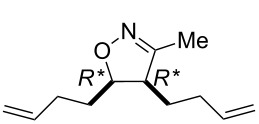


Yellow oil; yield 38%; *R*_f_ = 0.57 (*n*-hexane/EtOAc 4:1); ^1^H NMR (CDCl_3_, 400 MHz) δ 1.51–1.74 (m, 4H, CH_2_), 2.00 (s, 3H, CH_3_), 2.08–2.19 (m, 3H, CH_2_), 2.39–2.48 (m, 1H, CH_2_), 2.97–3.05 (m, 1H, H-4), 4.46–4.52 (m, 1H, H-5), 5.02–5.16 (m, 4H, CH=), 5.68–5.79 (m, 2H, CH=); ^13^C NMR (DMSO, 125 MHz) δ 12.6, 24.5, 27.6, 30.7, 32.0, 51.0, 81.7, 115.6, 115.8, 138.4, 138.6, 159.8; anal. calcd for C_12_H_19_NO: C, 74.57; H, 9.91; N, 7.25; found, C, 74.20; H, 9.65; N, 6.86.

(3S*,5R*)-3,5-Divinylpyrrolidin-2-one ((±)-**19).**


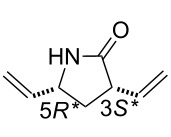


White solid; yield 70%; mp 67–68 °C; *R*_f_ = 0.40 (*n*-hexane/EtOAc 1:2); ^1^H NMR (CDCl_3_, 400 MHz) δ 1.65–1.72 (m, 1H, CH_2_), 2.47–2.53 (m, 1H, CH_2_), 3.09–3.18 (m, 1H, H-3), 4.05–4.13 (m, 1H, H-5), 5.13–5.25 (m, 4H, CH=), 5.74–5.81 (m, 1H, CH=), 5.84–5.92 (m, 1H, CH=), 6.00 (brs, 1H, NH); ^13^C NMR (CDCl_3_, 100 MHz) δ 34.9, 46.0, 55.2, 116.8, 117.7, 135.0, 138.5, 177.4; MS (ESI, pos) (*m*/*z*): 138 [M + 1]; anal. calcd for C_8_H_11_NO: C, 70.04; H, 8.08; N, 10.21; found, C, 69.69; H, 7.81; N, 9.86.

(3S*,5R*)-5-((*E*)-3-Oxobut-1-en-1-yl)-3-vinylpyrrolidin-2-one ((±)-**20).**


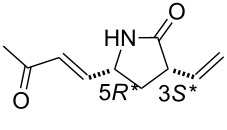


White solid; yield 28%; mp 58–89 °C; *R*_f_ = 0.45 (*n*-hexane/EtOAc 1:2); ^1^H NMR (CDCl_3_, 400 MHz) δ 1.75–1.82 (m,1H, CH_2_), 2.36 (s, 3H, CH_3_), 2.53–2.62 (m, 1H, CH_2_), 3.27–3.35 (m, 1H, H-3), 4.29–4.37 (m, 1H, H-5), 5.27–5.35 (m, 2H, CH=), 5.88–5.97 (m, 1H, CH=), 6.20–6.27 (d, *J* = 16.1 Hz, 1H, CH=), 6.51 (brs, 1H, NH), 6.60–6.68 (dd, *J* = 16.2 Hz, *J* = 6.6 Hz, 1H, CH=); ^13^C NMR (CDCl_3_, 100 MHz) δ 27.6, 34.1, 45.7, 53.4, 118.2, 130.5, 134.3, 145.3, 177.7, 197.7; MS (ESI, pos) (*m*/*z*): 181 [M + 1]; anal. calcd for C_10_H_13_NO_2_: C, 67.02; H, 7.31; N, 7.82; found, C, 67.33; H, 7.01; N, 7.52.

(R*,*Z*)-3-Ethylidene-5-vinylpyrrolidin-2-one ((±)-**23).**


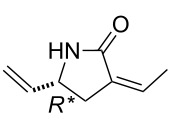


White solid; yield 69%; mp 49–50 °C; *R*_f_ = 0.35 (*n*-hexane/EtOAc 1:2); ^1^H NMR (CDCl_3_, 400 MHz) δ 1.77 (d, *J* = 6.9 Hz, 3H, CH_3_), 2.45–2.51 (m, 1H, CH_2_), 2.98–3.04 (m, 1H, CH_2_), 4.19–4.28 (m, 1H, H-5), 5.02–5.08 (d, *J* =10.1 Hz, 1H, CH=), 5.20–5.27 (d, *J* =16.6 Hz, 1H, CH=), 5.78–5.88 (m 1H, CH=), 6.48–6.54 (m, 1H, CH=), 7.51 (brs, 1H, NH); ^13^C NMR (CDCl_3_, 100 MHz) δ 14.8, 31.2, 53.9, 115.6, 128.6, 131.5, 139.2, 171.5; MS (ESI, pos) (*m*/*z*): 138 [M + 1]; anal. calcd for C_8_H_11_NO: C, 70.04; H, 8.08; N, 10.21; found, C, 69.70; H, 7.80; N, 9.84.

## Supporting Information

File 1 Copies of NMR spectra.
