# Supplementary material for: Ring-opening metathesis of some strained bicyclic systems; stereocontrolled access to diolefinated saturated heterocycles with multiple stereogenic centers
Source: Beilstein J Org Chem. 2018 Oct 24;14:2698–707. doi: 10.3762/bjoc.14.247 (PMC6204778; doi:10.3762/bjoc.14.247)
Supplement: File 1 — Copies of NMR spectra. [file Beilstein_J_Org_Chem-14-2698-s001.pdf]

Supporting Information

for

Ring-opening metathesis of some strained bicyclic systems; stereocontrolled access to diolefinated saturated heterocycles with multiple stereogenic centers

Zsanett Benke[1], Melinda Nonn[1,2], Márton Kardos[1], Santos Fustero[3] and Loránd Kiss[1*]

Address: [1]Institute of Pharmaceutical Chemistry, University of Szeged, H-6720 Szeged, Eötvös u. 6, Hungary, [2]MTA-SZTE Stereochemistry Research Group, Hungarian Academy of Sciences, H-6720 Szeged, Eötvös u. 6, Hungary and [3]Departamento de Química Orgánica, Facultad de Farmàcia, Universidad de Valencia, Av. Vicente Andrés Estellés, s/n 46100 Valencia, Spain

Email: Loránd Kiss - kiss.lorand@pharm.u-szeged.hu

*Corresponding author

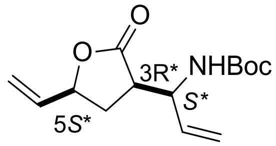
O
O
3R*
NHBoc
S*
5S*

(±)-**5**

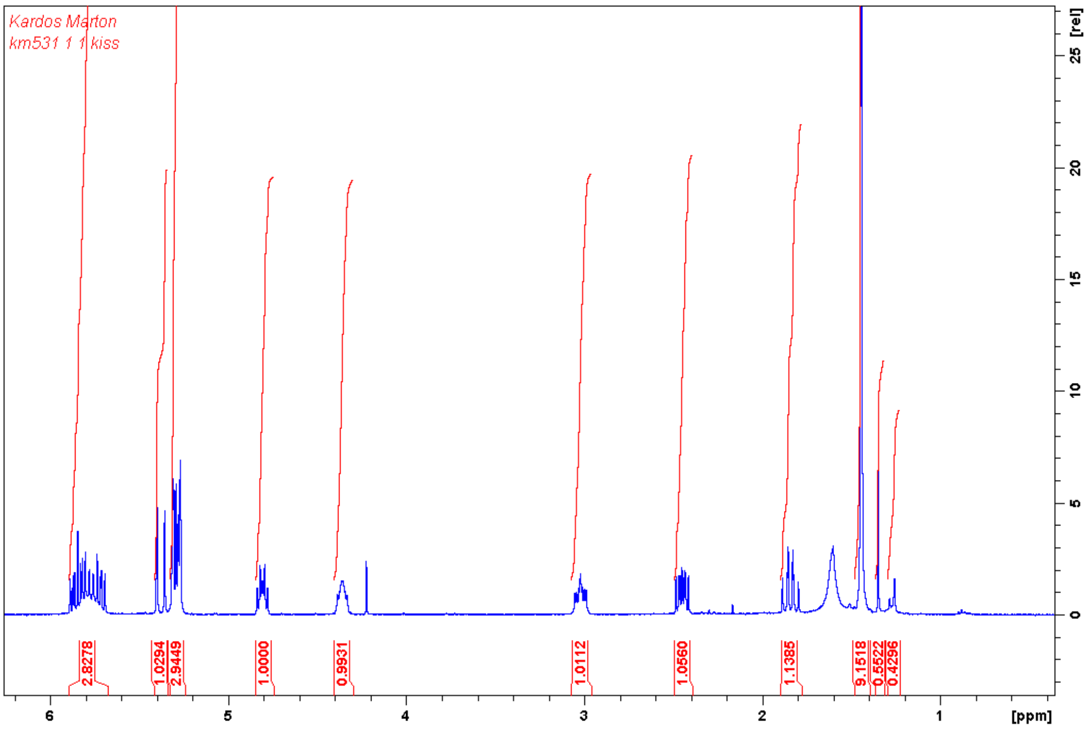
Kardos Marton
km531 1 1 kiss
2.8278
1.0294
2.9449
1.0000
0.9931
1.0112
1.0560
1.1385
9.1518
0.5522
0.4296
[ppm]
[rel]

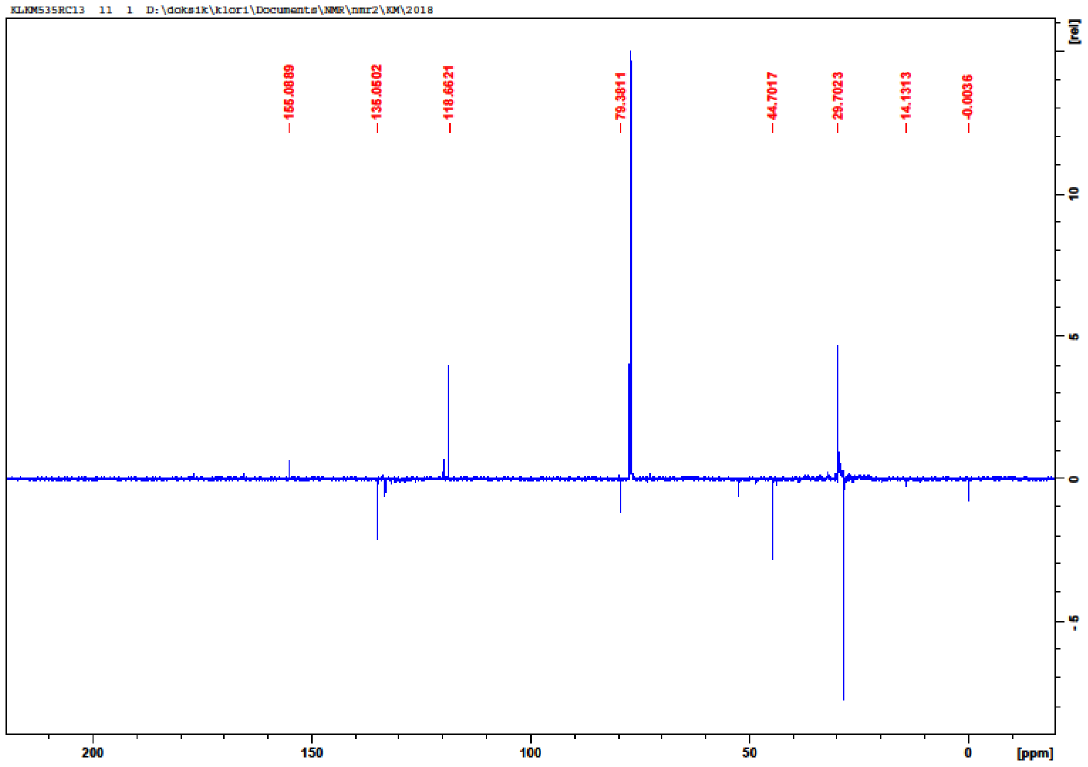
KLKM535RC13 11 1 D:\doksik\klori\Documents\NMR\nmr2\KM\2018
155.0889
135.0502
118.6621
79.3811
44.7017
29.7023
14.1313
-0.0036
[ppm]
[rel]

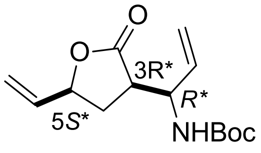
O
O
3R*
R*
5S*
NHBoc

(±)-**6**

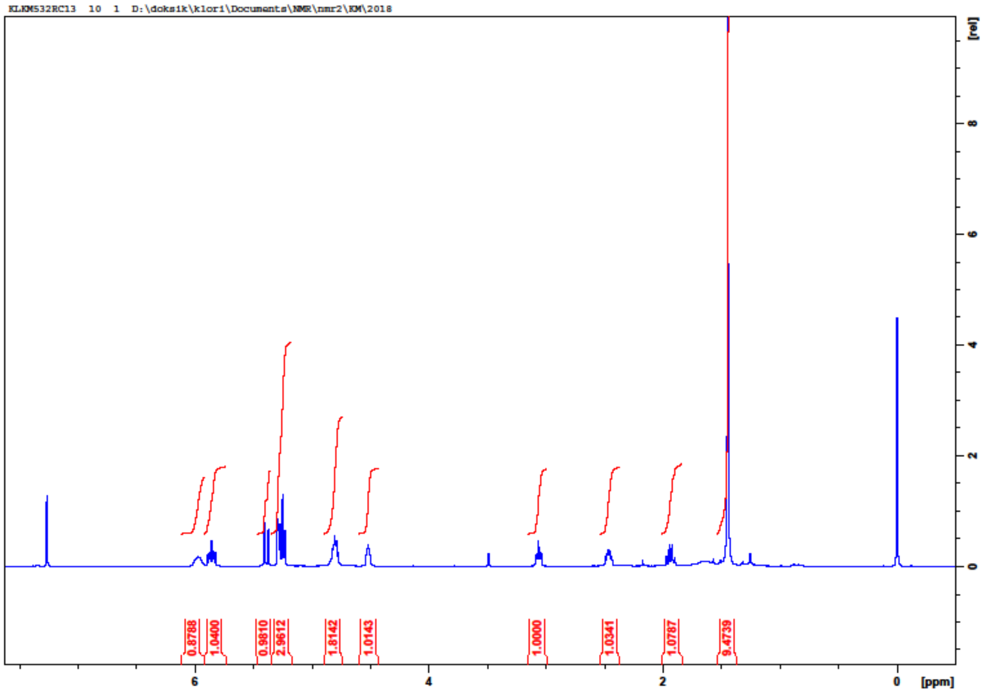
KLKM532RC13 10 1 D:\doksik\klori\Documents\NMR\nmr2\KM\2018
[rel]
0.8768
1.0400
0.9810
2.9612
1.8142
1.0143
1.0000
1.0341
1.0787
9.4739
[ppm]

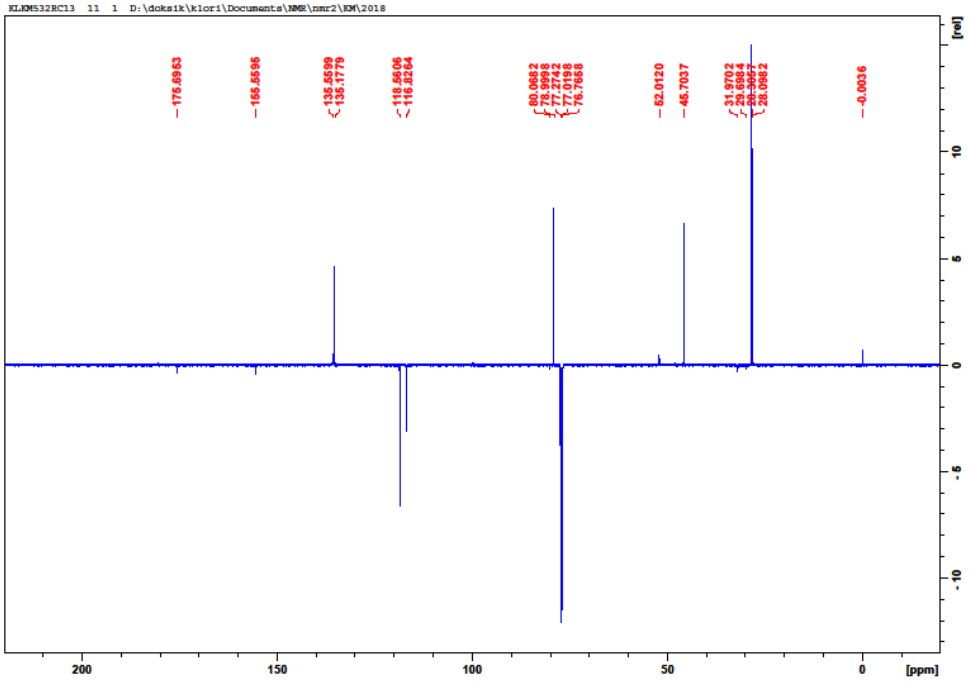
KLKM532RC13 11 1 D:\doksik\klori\Documents\NMR\nmr2\KM\2018
[rel]
175.6953
155.5595
135.5599
135.1779
118.5606
116.8264
80.0682
78.9998
77.2742
77.0198
76.7658
52.0120
45.7037
31.9702
29.6984
28.0982
-0.0036
[ppm]

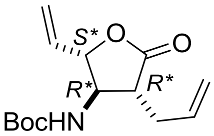

(±)-**10**

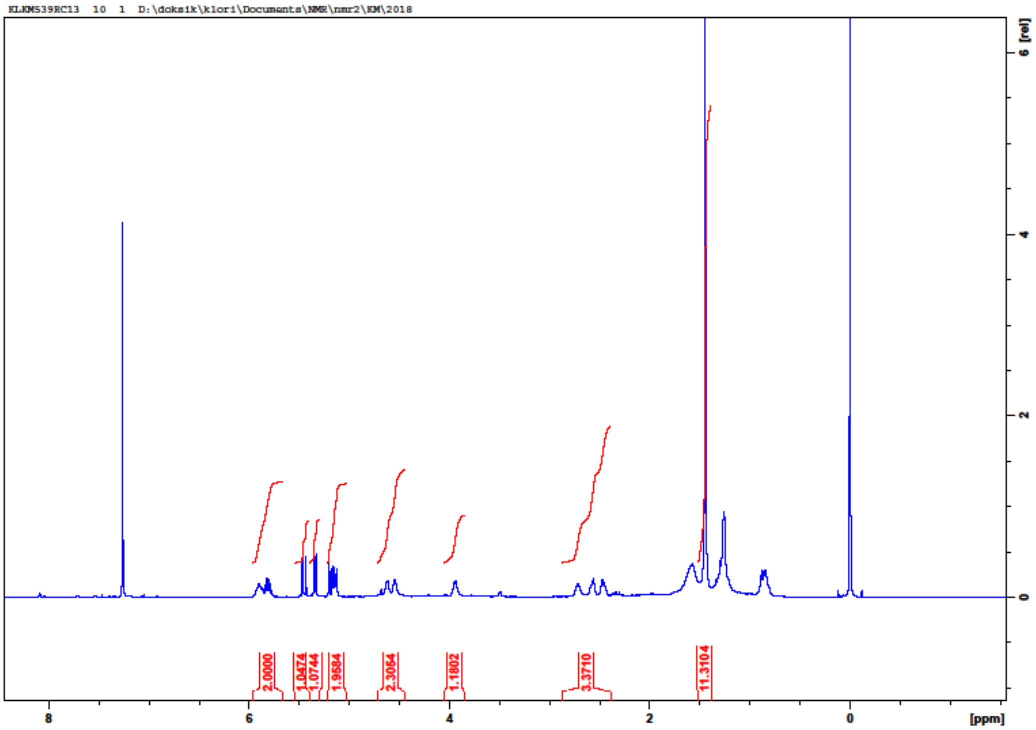
KLKM539RC13 10 1 D:\doksik\klori\Documents\NMR\nmr2\KM\2018
2.0000
1.0474
1.0744
1.9584
2.3054
1.1802
3.3710
11.3104
[ppm]
[rel]

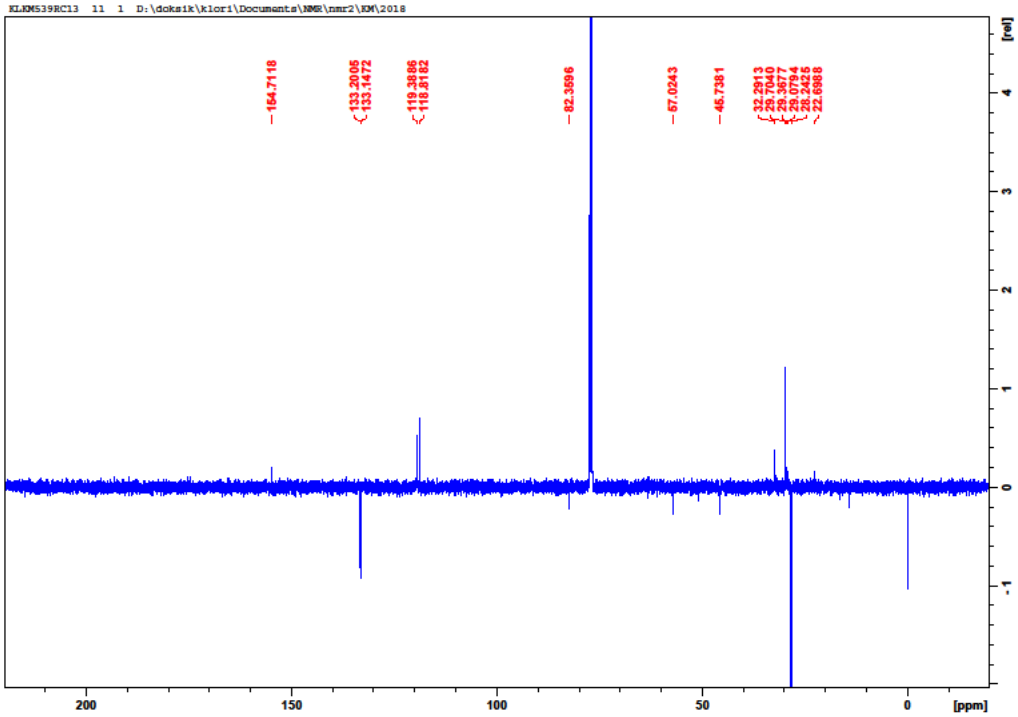
KLKM539RC13 11 1 D:\doksik\klori\Documents\NMR\nmr2\KM\2018
154.7118
133.2005
133.1472
119.3886
118.8182
82.3596
57.0243
45.7381
32.2913
29.7040
29.3677
29.0794
28.2425
22.6988
[ppm]
[rel]

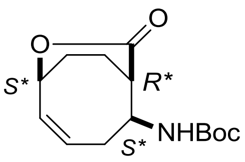
O
O
S*
R*
NHBoc
S*

(±)-**14**

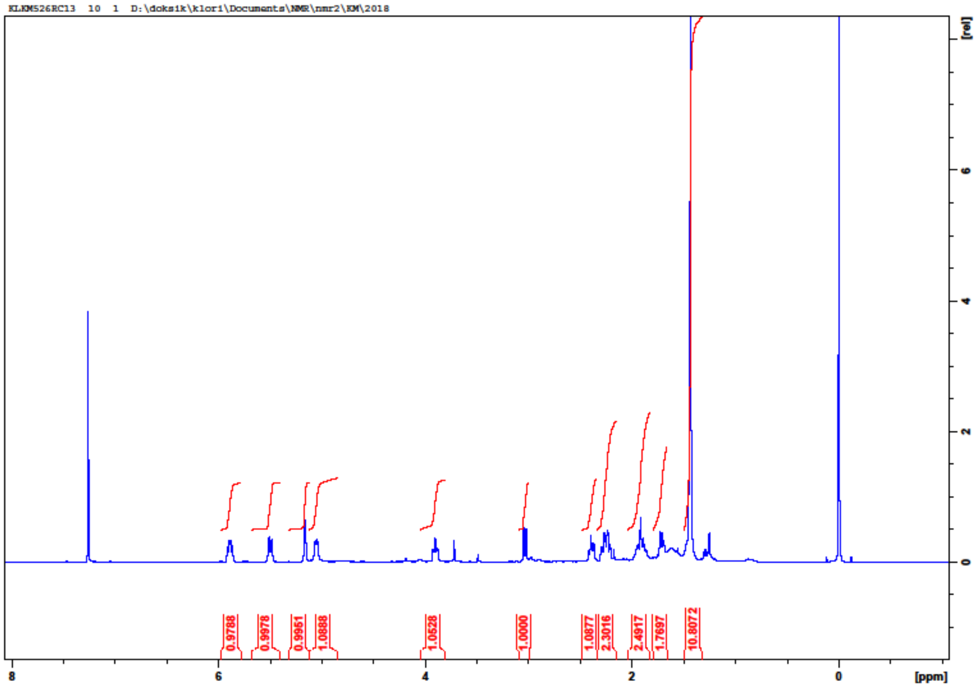
KLKM526RC13 10 1 D:\doksik\klori\Documents\NMR\nmr2\KM\2018
0.9788
0.9978
0.9961
1.0888
1.0528
1.0000
1.0877
2.3016
2.4917
1.7697
10.8072
8
6
4
2
0
[ppm]
[rel]

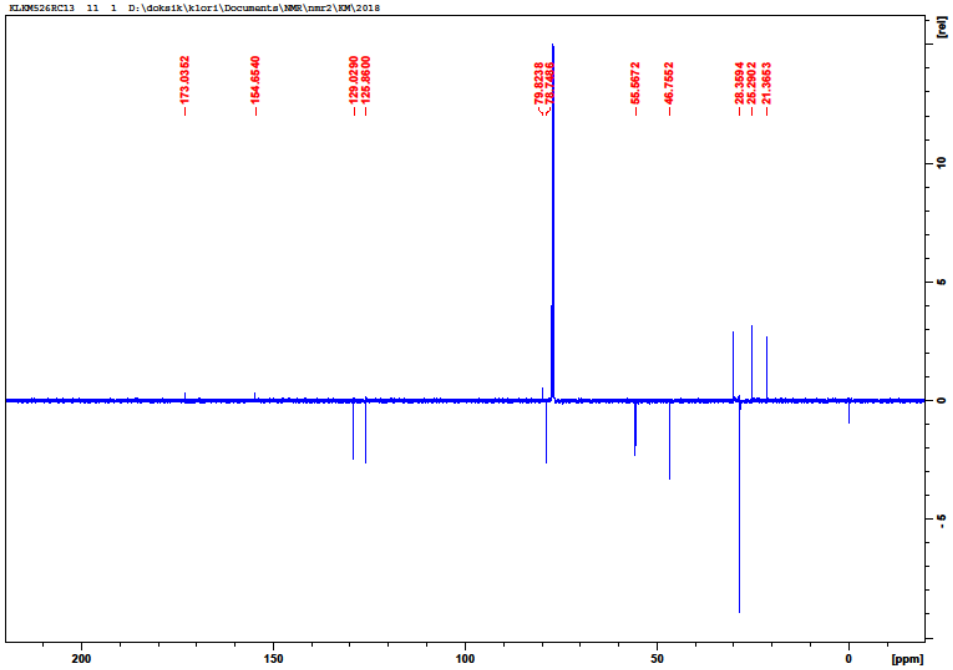
KLKM526RC13 11 1 D:\doksik\klori\Documents\NMR\nmr2\KM\2018
173.0352
154.6540
129.0290
126.8600
79.8238
78.7486
55.5672
46.7552
28.3594
25.2902
21.3653
200
150
100
50
0
[ppm]
[rel]

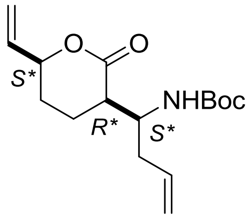
O
O
S*
R*
S*
NHBoc

(±)-**15**

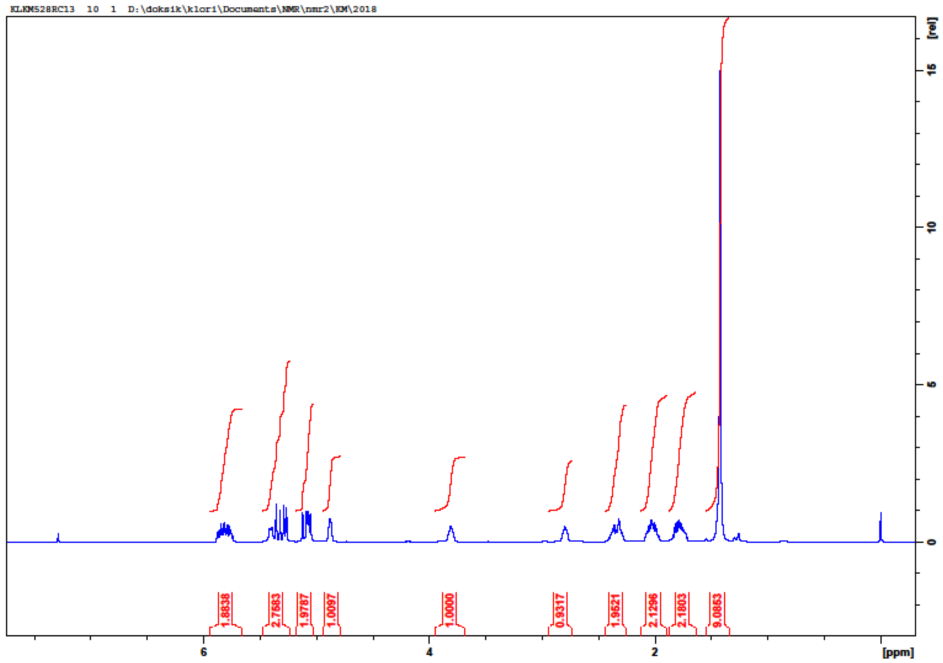
KLKM528RC13 10 1 D:\doksik\klori\Documents\NMR\nmr2\KM\2018
[rel]
1.8838
2.7583
1.9787
1.0097
1.0000
0.9317
1.9621
2.1296
2.1803
9.0853
[ppm]

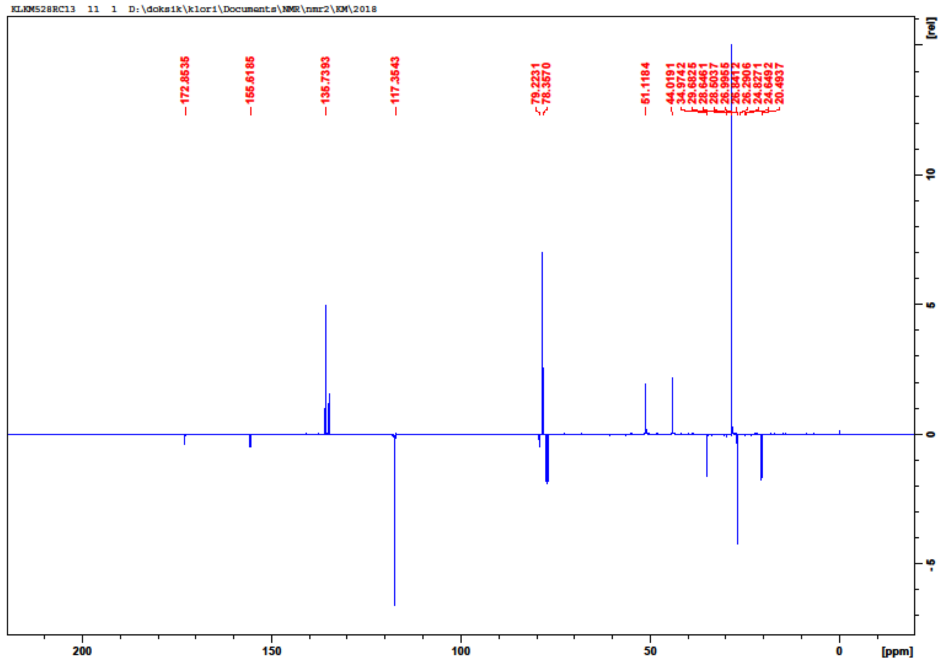
KLKM528RC13 11 1 D:\doksik\klori\Documents\NMR\nmr2\KM\2018
[rel]
172.8535
155.6185
135.7393
117.3543
79.2231
78.3570
51.1184
44.0191
34.9742
29.6825
28.6461
28.6037
26.9965
26.8412
26.2906
24.8271
24.6492
20.4937
[ppm]

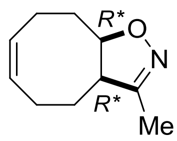

(±)-**16**

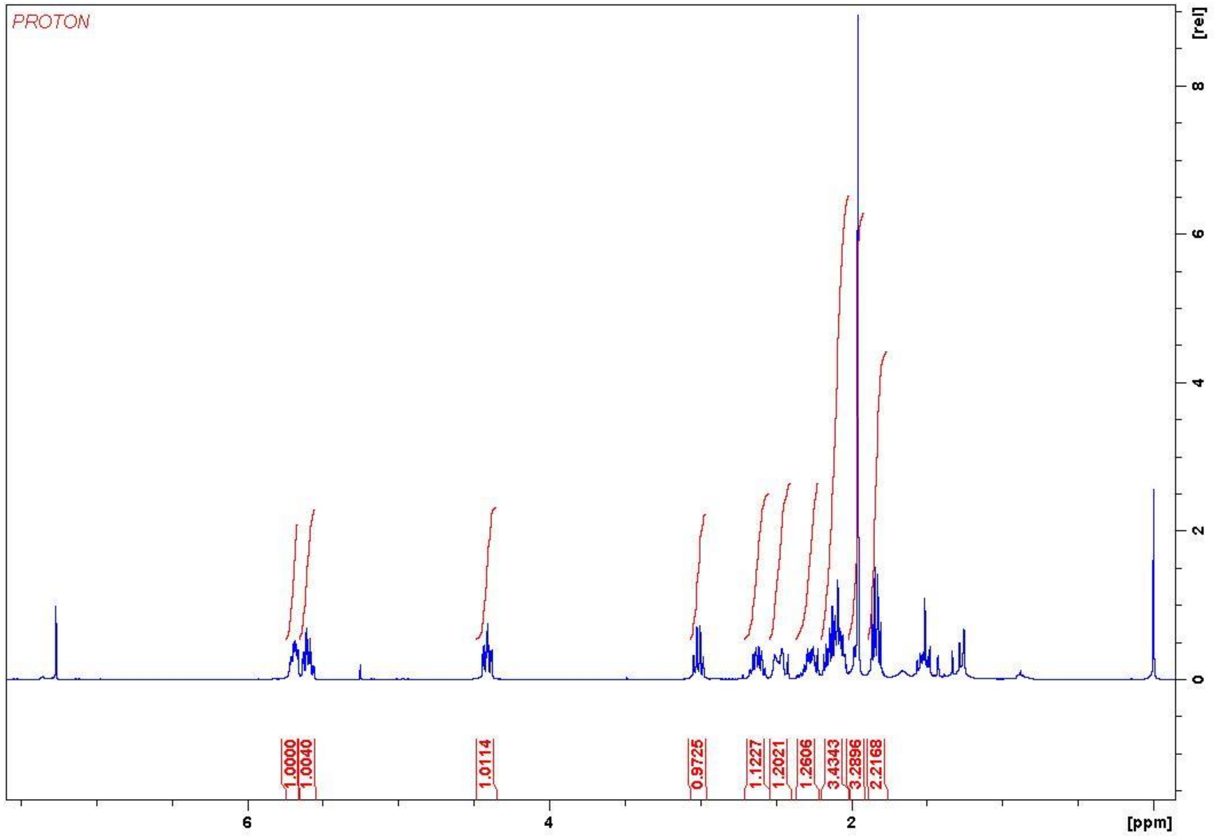

PROTON
[rel]
8
6
4
2
0
1.0000
1.0040
1.0114
0.9725
1.1227
1.2021
1.2606
3.4343
3.2896
2.2168
6
4
2
[ppm]

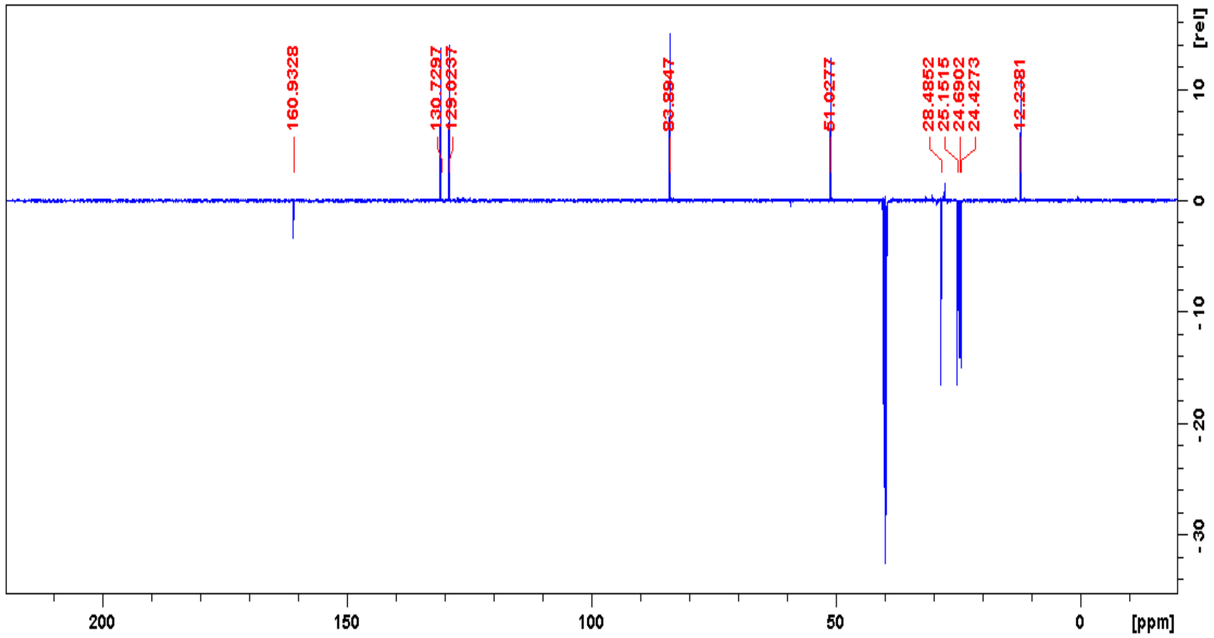

160.9328
130.7297
129.0237
83.8947
51.0277
28.4852
25.1515
24.6902
24.4273
12.2381
[rel]
10
0
-10
-20
-30
200
150
100
50
0
[ppm]

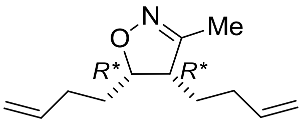

O
N
Me
R*
R*

(±)-**17**

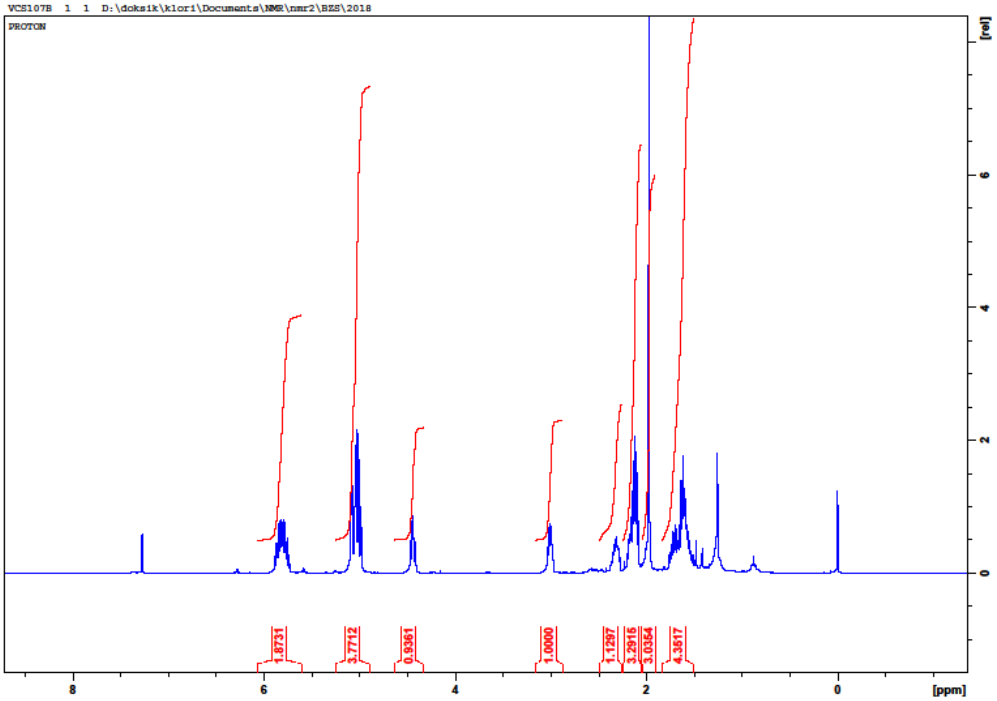

VCS107B 1 1 D:\doksik\klori\Documents\NMR\nmr2\BZS\2018
PROTON
[rel]
1.8731
3.7712
0.9361
1.0000
1.1297
3.2916
3.0354
4.3517
8
6
4
2
0
[ppm]

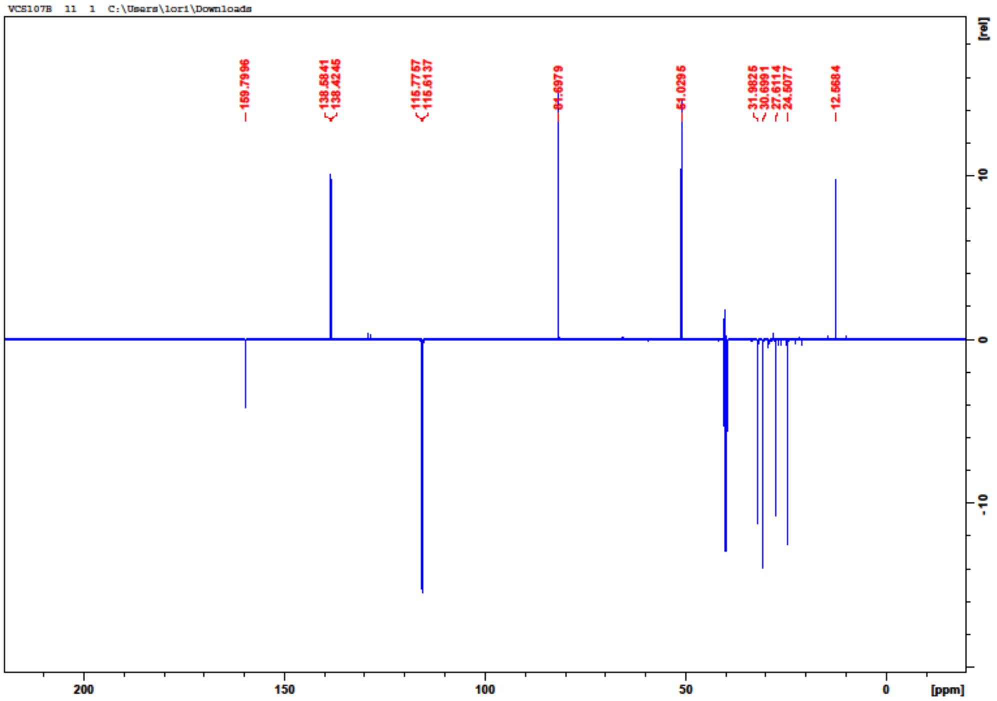

VCS107B 11 1 C:\Users\lori\Downloads
[rel]
159.7996
138.5841
138.4245
115.7757
115.6137
81.6979
51.0295
31.9825
30.6991
27.6114
24.5077
12.5684
200
150
100
50
0
[ppm]

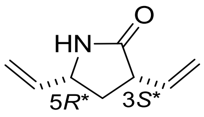
HN
O
5R*
3S*

(±)-**19**

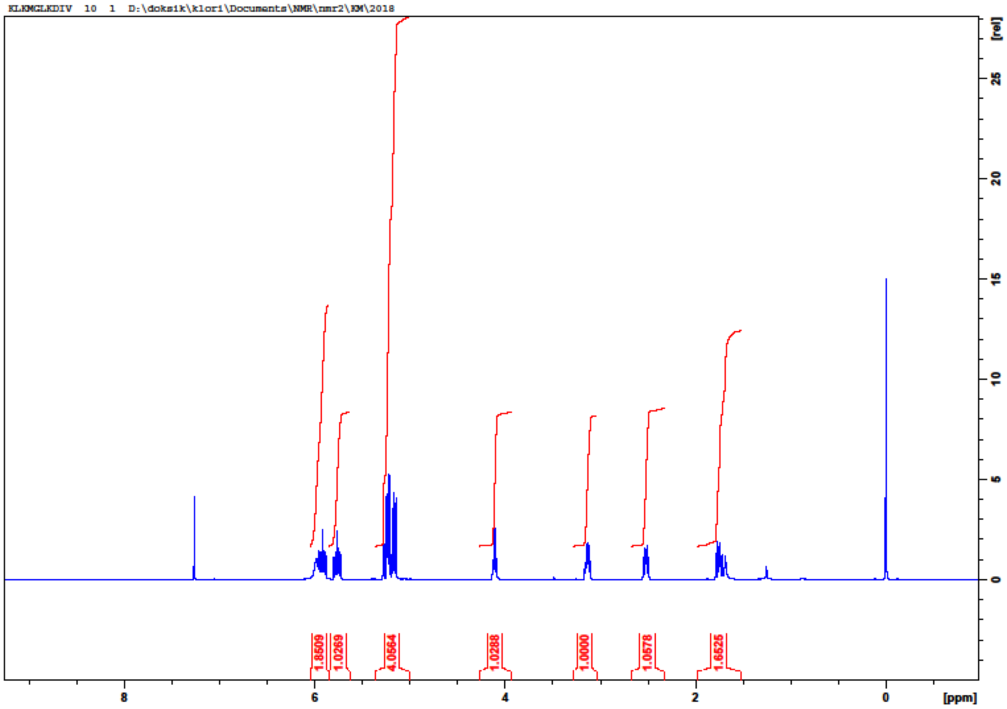
KLKMGLKDIV 10 1 D:\doksik\klori\Documents\NMR\nmr2\KM\2018
1.8509
1.0269
4.0564
1.0288
1.0000
1.0578
1.6525
[rel]
[ppm]

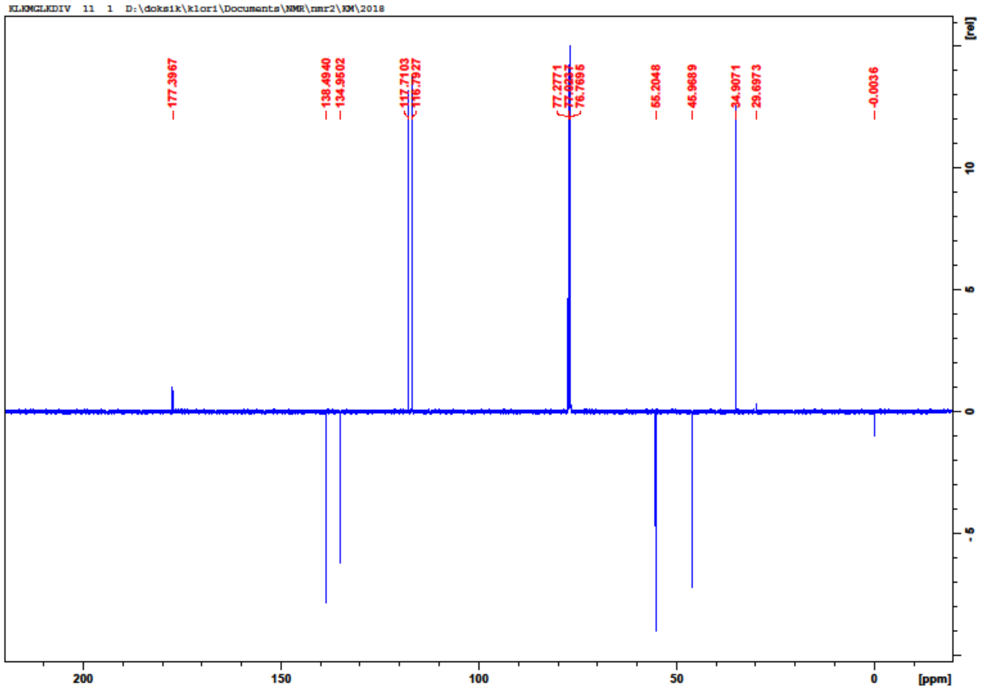
KLKMGLKDIV 11 1 D:\doksik\klori\Documents\NMR\nmr2\KM\2018
177.3967
138.4940
134.9502
117.7103
116.7927
77.2771
76.7696
55.2048
45.9689
34.9071
29.6973
-0.0036
[rel]
[ppm]

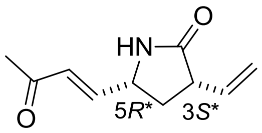
HN
O
O
5R*
3S*

(±)-**20**

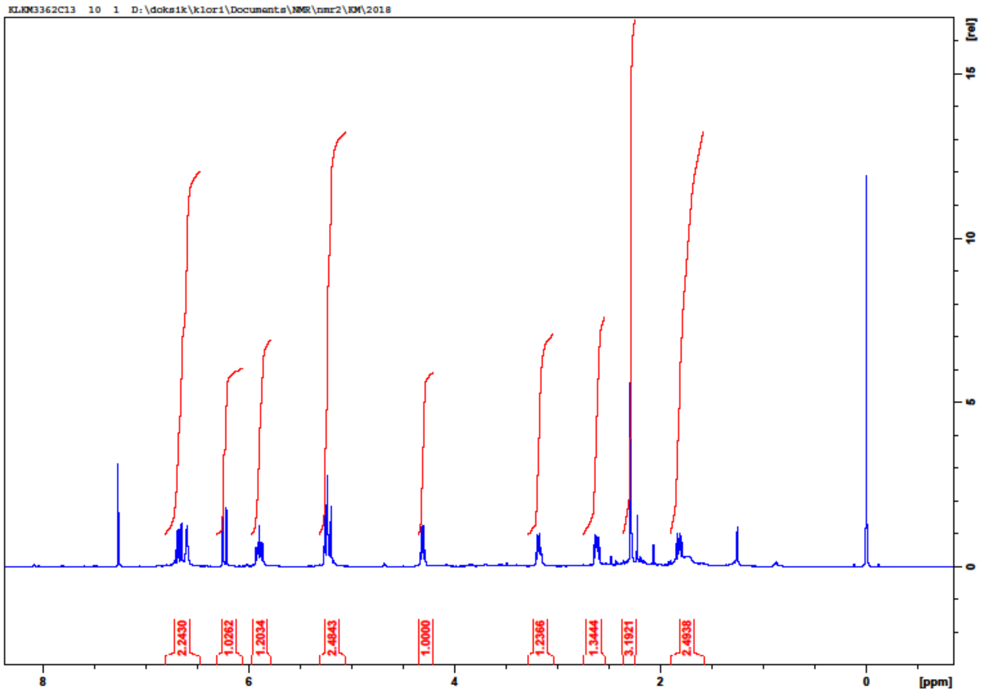
KLKM3362C13 10 1 D:\doksik\klori\Documents\NMR\nmr2\KM\2018
[rel]
2.2430
1.0262
1.2034
2.4843
1.0000
1.2366
1.3444
3.1921
2.4938
8
6
4
2
0
[ppm]

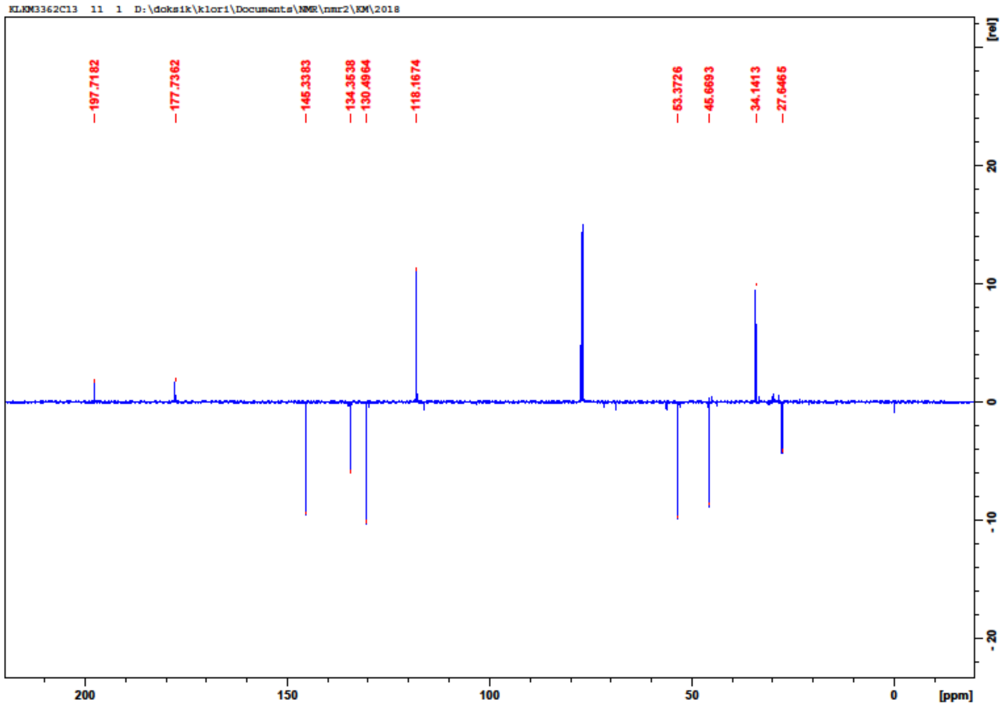
KLKM3362C13 11 1 D:\doksik\klori\Documents\NMR\nmr2\KM\2018
[rel]
197.7182
177.7362
145.3383
134.3538
130.4964
118.1674
53.3726
45.6693
34.1413
27.6465
200
150
100
50
0
[ppm]

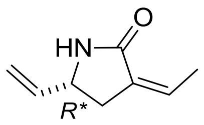
O
HN
R*

(±)-**23**

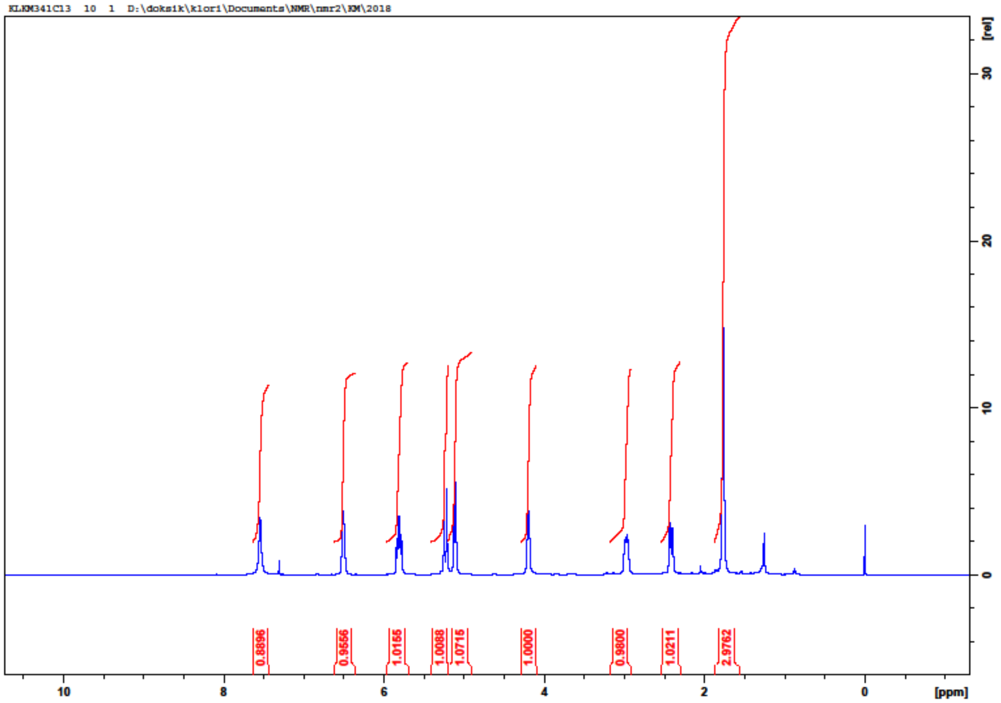
KLKM341C13 10 1 D:\doksik\klori\Documents\NMR\nmr2\KM\2018
[rel]
0.8896
0.9556
1.0155
1.0088
1.0716
1.0000
0.9800
1.0211
2.9762
10
8
6
4
2
0
[ppm]

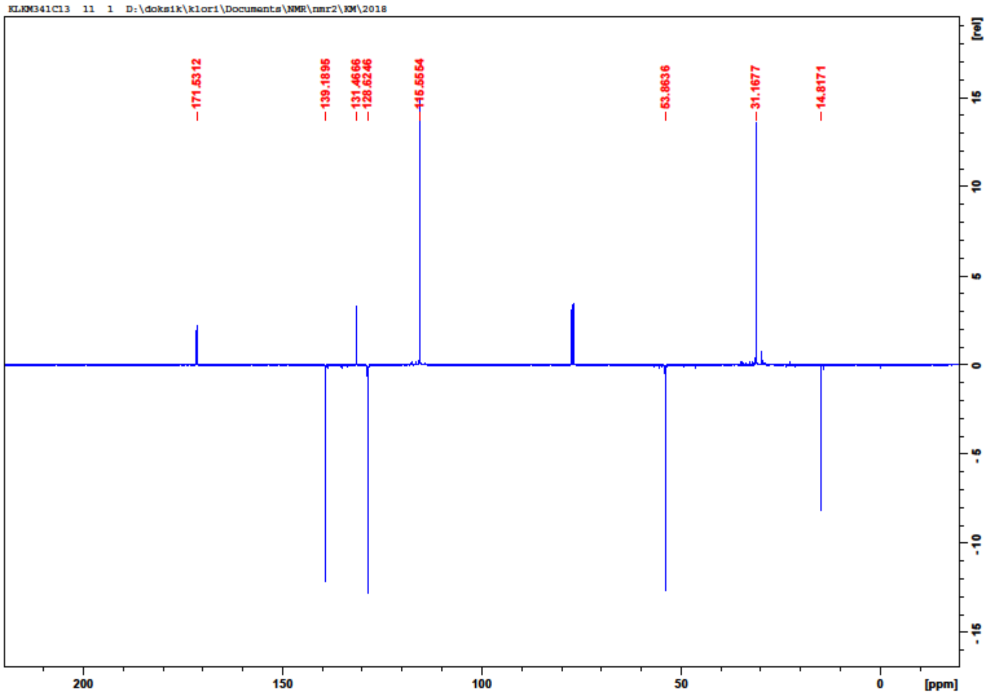
KLKM341C13 11 1 D:\doksik\klori\Documents\NMR\nmr2\KM\2018
[rel]
171.5312
139.1895
131.4666
128.6246
115.5554
53.8636
31.1677
14.8171
200
150
100
50
0
[ppm]
